# Swarm Intelligence and Its Applications 2014

**DOI:** 10.1155/2014/204294

**Published:** 2014-06-23

**Authors:** Yudong Zhang, Praveen Agarwal, Vishal Bhatnagar, Saeed Balochian, Xuewu Zhang

**Affiliations:** ^1^School of Computer Science and Technology, Nanjing Normal University, Nanjing, Jiangsu 210023, China; ^2^Department of Mathematics, Anand International College of Engineering, Agra Road, Near Bassi, Jaipur, Rajasthan 303012, India; ^3^Ambedkar Institute of Advanced Communication Technologies and Research, Government of NCT of Delhi, Geeta Colony, Delhi 110031, India; ^4^Department of Electrical Engineering, Gonabad Branch, Islamic Azad University, Gonabad, Khorasan-e-Razavi 96916-29, Iran; ^5^MRI Lab, Columbia University, New York, NY 10032, USA

Swarm intelligence (SI) represents the collective behavior of decentralized, self-organized systems. SI systems consist typically of a population of simple agents that interact locally with one another and with their environment. The inspiration of SI originates from biological systems. The agents follow very simple rules, and although there is no centralized control structure dictating how individual agents should behave, local, and to a certain degree random, interactions between such agents lead to the emergence of intelligence, unknown to the individual agents. Natural examples of SI include ant colonies, bird flocking, animal herding, bacterial growth, and fish schooling. Besides the applications to conventional optimization problems, SI is employed in various fields such as library materials acquisition, communications, medical dataset classification, dynamic control, heating system planning, moving objects tracking, pattern recognition, and statistical prediction.

The main objective of this special issue is to provide the readers with a collection of high quality research articles that address the broad challenges in application aspects of swarm intelligence and reflect the emerging trends in state-of-the-art algorithms.

The paper authored by Z.-C. Wang and X.-B. Wu (Tongji University) investigates the applicability and performance of biogeography-based optimization (BBO) for integer programming. They find that the original BBO algorithm does not perform well on a set of benchmark problems of integer programming. Hence, they modify the mutation operator and/or the neighborhood structure of the algorithm, resulting in three new BBO-based methods, named BlendBBO, BBO_DE, and LBBO_LDE, respectively. Computational experiments show that these methods are competitive approaches to solve integer-programming problems, and the LBBO_LDE shows the best performance on the benchmark problems.

In the paper by J. Wang et al. (North China Electric Power University), they model the complex process-planning problem as a combinatorial optimization problem with constraints. An ant colony optimization (ACO) approach is developed to deal with process planning problem by simultaneously considering activities such as sequencing operations, selecting manufacturing resources, and determining setup plans to achieve the optimal process plan. A weighted directed graph is conducted to describe the operations, precedence constraints between operations, and the possible visited path between operation nodes. A representation of process plan is described based on the weighted directed graph. Ant colony goes through the necessary nodes on the graph to achieve the optimal solution with the objective of minimizing total production costs. Two cases are carried out to study the influence of various parameters of ACO on the system performance. Extensive comparative experiments are conducted to demonstrate the feasibility and efficiency of the proposed approach.

R. Kalatehjari et al. (Universiti Teknologi Malaysia) apply particle swarm optimization (PSO) in three-dimensional (3D) slope stability problem to determine the critical slip surface (CSS) of soil slopes. A detailed description of adopted PSO is presented to provide a good basis for more contribution of this technique to the field of 3D slope stability problems. A general rotating ellipsoid shape is introduced as the specific particle for 3D slope stability analysis. A detailed sensitivity analysis is designed and performed to find the optimum values of parameters of PSO. Example problems are used to evaluate the applicability of PSO in determining the CSS of 3D slopes. The first example presents a comparison between the results of PSO and PLAXI-3D finite element software. The second example compares the ability of PSO to determine the CSS of 3D slopes with other optimization methods from the literature. The results demonstrate the efficiency and effectiveness of PSO in determining the CSS of 3D soil slopes.

Another paper is by Y. Sun et al. (Beijing University of Posts and Telecommunications, Columbia University). It focuses on how to outsource computation task to the cloud securely and proposes a secure outsourcing multiparty computation protocol on lattice-based encrypted data in two-cloud-server scenario. The main idea is to transform the outsourced data, respectively, encrypted by different users' public keys to the ones encrypted by the same two private keys of the two assisted servers, so that it is feasible to operate on the transformed cipher-texts to compute an encrypted result following the function to be computed. In order to keep the privacy of the result, the two servers cooperatively produce a custom-made result for each user that is authorized to get the result, so that all authorized users can recover the desired result while other unauthorized ones including the two servers cannot. Compared with previous research, the protocol is completely noninteractive between any users. Both of the computation and the communication complexities of each user in their solution are independent of the computing function.

In their paper, M.-Y. Ju et al. (National University of Tainan) propose a hybrid evolutionary algorithm using scalable encoding method for path planning problems. The scalable representation is based on binary tree structure encoding. To solve the problem of hybrid genetic algorithm and particle swarm optimization, the “dummy node” is added to the binary trees to deal with the different lengths of representations. The experimental results show that the proposed hybrid method uses fewer turning points than traditional evolutionary algorithms and generate shorter collision-free paths for mobile robot navigation.

The paper by H. Mo et al. (Harbin Engineering University, Harbin University of Commerce, University of Pretoria, and Shaoxing University) proposes a novel constrained multiobjective biogeography optimization algorithm (CMBOA). It is the first biogeography optimization algorithm for constrained multiobjective optimization. In CMBOA, a disturbance migration operator is designed to generate diverse feasible individuals, in order to promote the diversity of individuals on Pareto front. Infeasible individuals nearby feasible region are evolved to feasibility by recombining with their nearest nondominated feasible individuals. The convergence of CMBOA is proved by using probability theory. The performance of CMBOA is evaluated on a set of 6 benchmark problems. The experimental results show that the CMBOA performs better than or similar to the classical NSGA-II and IS-MOEA.

The paper authored by I. C. Obagbuwa and A. O. Adewumi (University of KwaZulu-Natal) introduces the hunger component to the existing cockroach swarm optimization (CSO) algorithm, to improve its searching ability and population diversity. The original CSO is modelled with three components: chase-swarming, dispersion, and ruthlessness; additional hunger component modelled using partial differential equation (PDE) method is included. The performance of the proposed algorithm is tested on well-known benchmarks and compared with the existing CSO, modified cockroach swarm optimization (MCSO), roach infestation optimization RIO, and hungry roach infestation optimization (HRIO). The comparison results show clearly that the proposed algorithm outperforms the existing algorithms.

In the paper by L. Liu et al. (Harbin Engineering University), they propose a distribution model of ant colony foraging, through analysis of the relationship between the position distribution and food source in the process of ant colony foraging. They design a continuous domain optimization algorithm based on the model. They give the form of solution for the algorithm, the distribution model of pheromone, the update rules of ant colony position, and the processing method of constraint condition. The algorithm is tested against a set of test trials by unconstrained optimization test functions and a set of optimization test functions. The results of other algorithms are compared and analyzed, to verify the correctness and effectiveness of the proposed algorithm.

A. Shabri and R. Samsudin (Universiti Teknologi Malaysia) propose a hybrid model integrating wavelet and multiple linear regressions (MLR) for crude oil price forecasting. In this model, Mallat wavelet transform is first selected to decompose an original time series into several subseries with different scale. Then, the principal component analysis (PCA) is used in processing subseries data in MLR for crude oil price forecasting. The particle swarm optimization (PSO) is used to adopt the optimal parameters of the MLR model. To assess the effectiveness of this model, daily crude oil market and West Texas Intermediate (WTI) are used as the case study. Time-series prediction capability performance of the WMLR model is compared with the MLR, ARIMA, and GARCH models using various statistics measures. The experimental results show that the proposed model outperforms the individual models in forecasting of the crude oil prices series.

In their paper, F. A. Ahmad et al. (Universiti Putra Malaysia) propose a new approach based on integrated intelligent system inspired by foraging of honeybees applied to multimobile robot scenario. This integrated approach caters for both working and foraging stages for known/unknown power station locations. Swarm mobile robot inspired by honeybee is simulated to explore and identify the power station for battery recharging. The mobile robots will share the location information of the power station with each other. The results show that mobile robots consume less energy and less time when they are cooperating with each other for foraging process. The optimizing of foraging behavior will result in the mobile robots spending more time to do real work.

The paper by J.-Q. Li et al. (Northeastern University and Liaocheng University) proposes a hybrid algorithm that combines particle swarm optimization (PSO) and iterated local search (ILS) for solving the hybrid flow-shop scheduling (HFS) problem with preventive maintenance (PM) activities. In the proposed algorithm, different crossover operators and mutation operators are investigated. In addition, an efficient multiple insert mutation operator is developed for enhancing the searching ability of the algorithm. Furthermore, an ILS-based local search procedure is embedded in the algorithm to improve the exploitation ability of the proposed algorithm. The detailed experimental parameter for the canonical PSO is tuning. The proposed algorithm is tested on the variation of 77 Carlier and Néron's benchmark problems. Detailed comparisons with the present efficient algorithms, including hGA, ILS, PSO, and IG, verify the efficiency and effectiveness of the proposed algorithm.

The paper authored by Q. Xu et al. (Shandong University) proposes a fast elitism Gaussian estimation of distribution algorithm (FEGEDA). The Gaussian probability model is used to model the solution distribution. The parameters of Gaussian come from the statistical information of the best individuals by fast learning rule, which enhances the efficiency of the algorithm. An elitism strategy is used to maintain the convergent performance. The performances of the algorithm are examined based upon several benchmarks. In the simulations, a one-dimensional benchmark is used to visualize the optimization process and probability model learning process during the evolution, and several two-dimensional and higher dimensional benchmarks are used to testify the performance of FEGEDA. The experimental results indicate the capability of FEGEDA, especially in the higher dimensional problems, and the FEGEDA exhibits a better performance than some other algorithms and EDAs. Finally, FEGEDA is used in PID controller optimization of PMSM and is compared with the classical PID and GA.

In the paper by K. S. Lim et al. (Universiti Teknologi Malaysia, Universiti Malaysia Pahang, Universiti Malaya, and Hanbat National University), their research incorporates the concept of multiple nondominated leaders to further improve the vector evaluated particle swarm optimization (VEPSO) algorithm. Multiple nondominated solutions that are best at a respective objective function are used to guide particles in finding optimal solutions. The improved VEPSO is measured by the number of nondominated solutions found, generational distance, spread, and hypervolume. The results from the conducted experiments show that the proposed VEPSO significantly improves the existing VEPSO algorithms.

Z. Yin et al. (Harbin Institute of Technology) focus on multiuser detection in tracking and data relay satellite (TDRS) system forward link. Minimum mean square error (MMSE) is a low complexity multiuser detection method, but MMSE detector cannot achieve satisfactory bit error ratio and near-far resistance, whereas artificial fish swarm algorithm (AFSA) is expert in optimization and it can realize the global convergence efficiently. Therefore, a hybrid multiuser detector based on MMSE and AFSA (MMSE-AFSA) is proposed. The result of MMSE and its modified formations are used as the initial values of artificial fishes to accelerate the speed of global convergence and reduce the iteration times for AFSA. The simulation results show that the bit error ratio and near-far resistance performances of the proposed detector are much better, compared with MF, DEC, and MMSE, and are quite close to OMD. Furthermore, the proposed MMSE-AFSA detector also has a large system capacity.

In their paper, S. Molla-Alizadeh-Zavardehi et al. (Islamic Azad University and University of Tehran) deal with a problem of minimizing total weighted tardiness of jobs in a real-world single batch-processing machine (SBPM) scheduling in the presence of fuzzy due date. First, a fuzzy mixed integer linear programming model is developed. Then, due to the complexity of the problem that is NP hard, they design two hybrid metaheuristics called GA-VNS and VNS-SA applying the advantages of genetic algorithm (GA), variable neighborhood search (VNS), and simulated annealing (SA) frameworks. Besides, they propose three fuzzy earliest due date heuristics to solve the given problem. Through computational experiments with several random test problems, a robust calibration is applied on the parameters. Finally, computational results on different-scale test problems are presented to compare the proposed algorithms.

The paper by H. Liu et al. (Beijing Institute of Technology, University of Science and Technology Liaoning, and Nanchang University) presents a human behavior-based PSO, which is called HPSO. There are two remarkable differences between PSO and HPSO. First, the global worst particle is introduced into the velocity equation of PSO, which is endowed with random weight that obeys the standard normal distribution; this strategy is conducive to trade off exploration and exploitation ability of PSO. Second, the two acceleration coefficients *c*_1_ and *c*_2_ in the standard PSO (SPSO) are eliminated to reduce the parameters sensitivity of solved problems. Experimental results on 28 benchmark functions, which consist of unimodal, multimodal, rotated, and shifted high-dimensional functions, demonstrate the high performance of the proposed algorithm in terms of convergence accuracy and speed with lower computation cost.

The paper authored by B. Crawford et al. (Pontificia Universidad Católica de Valparaíso, Universidad Finis Terrae, Universidad Autónoma de Chile, and Universidad Diego Portales) presents a novel application of the artificial bee colony algorithm to solve the nonunicost set covering problem. The artificial bee colony algorithm is a recent swarm metaheuristic technique based on the intelligent foraging behavior of honey bees. Experimental results show that the artificial bee colony algorithm is competitive in terms of solution quality with other recent metaheuristic approaches for the set covering problem.

In the paper by J.-S. Wang et al. (University of Science & Technology Liaoning), they propose an echo state network (ESN) based fusion soft-sensor model optimized by the improved glowworm swarm optimization (GSO) algorithm, for predicting the key technology indicators (concentrate grade and tailings recovery rate) of flotation process. Firstly, the color feature (saturation and brightness) and texture features (angular second moment, sum entropy, inertia moment, etc.) based on grey-level cooccurrence matrix (GLCM) are adopted to describe the visual characteristics of the flotation froth image. Then, the kernel principal component analysis (KPCA) method is used to reduce the dimensionality of the high-dimensional input vector composed by the flotation froth image characteristics and process datum and extracts the nonlinear principal components in order to reduce the ESN dimension and network complex. The ESN soft-sensor model of flotation process is optimized by the GSO algorithm with congestion factor. Simulation results show that the model has better generalization and prediction accuracy to meet the online soft-sensor requirements of the real-time control in the flotation process.

B. Li et al. (Shandong University and Qilu University of Technology) propose a novel KELM learning algorithm using the PSO approach to optimize the parameters of kernel functions of neural networks, which is called the AKELM learning algorithm, for improving the prediction accuracy of robot execution failures. The simulation results with the robot execution failures datasets show that, by optimizing the kernel parameters, the proposed algorithm has good generalization performance and outperforms KELM and the other approaches in terms of classification accuracy. Other benchmark problems simulation results also show the efficiency and effectiveness of the proposed algorithm.

In the paper by T. S. Kiong et al. (Universiti Tenaga Nasional, Universiti Kebangsaan Malaysia), their research considers the adaptive beamforming technique used to cancel interfering signals (placing nulls) and produce or steer a strong beam toward the target signal according to the calculated weight vectors. Minimum variance distortion response (MVDR) beamforming is capable of determining the weight vectors for beam steering; however, its nulling level on the interference sources remains unsatisfactory. Beamforming can be considered as an optimization problem, such that optimal weight vector should be obtained through computation. Hence, in their paper, a new dynamic mutated artificial immune system (DM-AIS) is proposed to enhance MVDR beamforming for controlling the null steering of interference and increase the signal to interference-noise ratio (SINR) for wanted signals.

Finally, F. Zou et al. (Xi'an University of Technology, Huaibei Normal University) present a new teaching-learning-based optimization (TLBO) variant called barebones teaching-learning-based optimization (BBTLBO), to solve the global optimization problems. In their method, each learner of teacher phase employs an interactive learning strategy, which is the hybridization of the learning strategy of teacher phase in the standard TLBO and Gaussian sampling learning based on neighborhood search, and each learner of learner phase employs the learning strategy of learner phase in the standard TLBO or the new neighborhood search strategy. To verify the performance of their approaches, 20 benchmark functions and 2 real-world problems are utilized. Conducted experiments can be observed that the BBTLBO performs significantly better than, or at least comparable to, TLBO and some existing bare-bone algorithms. The results indicate that the proposed algorithm is competitive to some other optimization algorithms. We expect that this special issue offers a comprehensive and timely view of the area of applications of SI and that it will offer stimulation for further research.

